# Unaccounted Gallbladder

**DOI:** 10.7759/cureus.65490

**Published:** 2024-07-27

**Authors:** Sunil Vishwakarma, Chhavindar Singh, Tripti Agarwal, Aditya Sagar

**Affiliations:** 1 General Surgery, Motilal Nehru Medical College, Prayagraj, IND

**Keywords:** acute calculous cholecystitis, chronic calculous cholecystitis, acalculous cholecystitis, cancer gall bladder, gall bladder dysmotility, pancreatitis, sphincter of oddi, agenesis of gall bladder, choledocholithiasis, cholelithiasis

## Abstract

Gallbladder agenesis is a rare anatomical variant, and most cases are asymptomatic and diagnosed on autopsy. Few of them may present with features suggestive of biliary tract pathology.

A 32-year-old male presented with complaints of intermittent epigastric pain for three months. Abdominal ultrasonography was suggestive of chronic calculous cholecystitis, and he was planned for laparoscopic cholecystectomy. However, no gallbladder was found during the surgery. Postoperative evaluation was suggestive of an absent gallbladder with a normal ductal system. A provisional diagnosis of sphincter of Oddi dysfunction was made based on his symptoms.

Congenital absence of gallbladder is a rare anomaly and only a few of the affected individuals are symptomatic. Lack of specific features, coupled with the inability of standard abdominal ultrasonography to detect the absence of gallbladder, can put the treating surgeon in a dilemma intraoperatively. Agenesis of the gallbladder is often missed and this entity should be kept in mind while having difficulty in visualizing the gallbladder. An astute surgeon should be wary of this diagnosis during difficult dissection to avoid bile duct injuries.

## Introduction

Agenesis of the gallbladder is uncommon and is characterized by the absence of a gallbladder with a normal bile duct system. It has an overall incidence of <0.1% (0.04-0.1%) [1] with a female preponderance in symptomatic cases (3:1) [2-3] and equivalent gender distribution in autopsy cases. The median age of diagnosis is 46 years. Few patients have symptoms, such as right upper quadrant abdominal pain (90%), nausea, vomiting (66%), intolerance to fatty food (37%), dyspepsia (30%) and jaundice (35%) [4]. It has been suggested that the pathophysiology of symptoms in gallbladder agenesis is similar to that of the post-cholecystectomy syndrome and that the causes of pain include biliary dyskinesia and choledocholithiasis.

The liver, gallbladder, and biliary system begin to develop early in the fourth week of intrauterine life as a ventral outgrowth from the caudal part of the foregut. This hepatic diverticulum divides into two parts as it grows, one representing the primordium of the liver, and the other, the primordium of the gallbladder and cystic duct. By the seventh week, from the pars cystic, a vacuole and a stalk develop, which respectively represent the gallbladder and the cystic duct. In the first stage, the gallbladder is a hollow organ, even if the proliferation of its epithelium determines a phase in which its cavity is temporarily cancelled; subsequently, through the vacuolation of its epithelium, it again becomes a hollow organ. Failure of this developmental process at any stage results in agenesis of the gallbladder [5,6]. It can be associated with gastrointestinal, cardiovascular, genitourinary, and skeletal malformations, such as duodenal atresia, malrotation of the gut, pancreas divisum, imperforate anus, hypoplasia of the right hepatic lobe, duplication cysts of the hepatic flexure, ventricular septal defect, renal agenesis, undescended testes, and syndactyly [7]. Routine imaging is unable to diagnose gallbladder agenesis leading to unnecessary operative intervention. It usually presents as an intraoperative surprise to the surgeon.

## Case presentation

**Figure 1:**
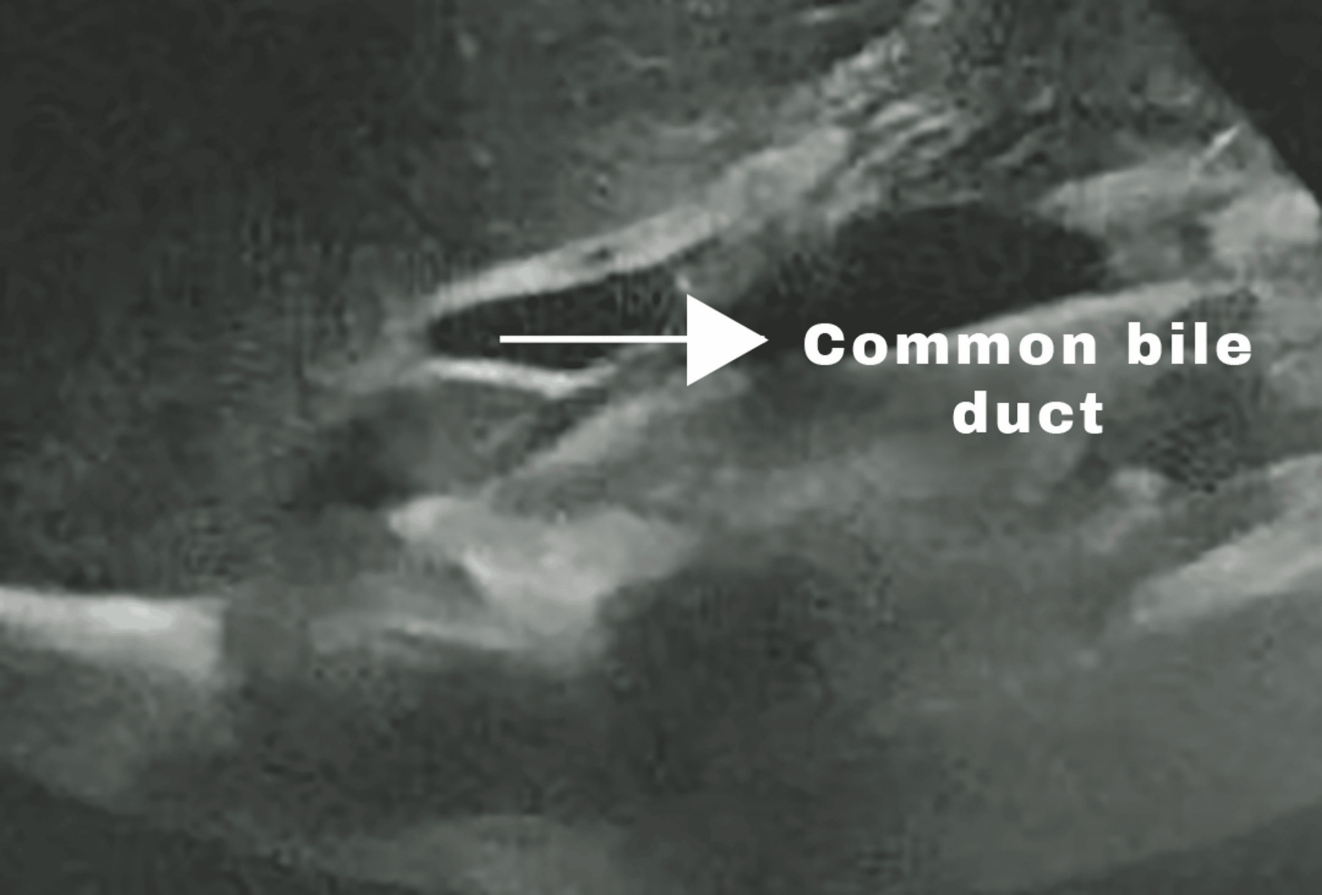
Pre-operative ultrasound abdomen showing normal common bile duct

A 32-year-old gentleman presented with complaints of intermittent nausea and pain over the epigastrium for four to three months. Clinical examination revealed tenderness over the epigastrium and right hypochondrium. Ultrasonography of the abdomen revealed a contracted gallbladder with a normal wall and a few calculi, the largest being 5.1 mm (Figure 1).

A provisional diagnosis of chronic calculous cholecystitis was made.

Routine blood investigations including complete blood count and liver function tests were within normal limits (Table 1) and the patient was planned for laparoscopic cholecystectomy after obtaining informed consent.

**Table 1:** Pre- and post-operative blood investigations

PRE-OPERATIVE INVESTIGATIONS		
INVESTIGATION	VALUE	REFERENCE RANGE
Hemoglobin	13.4 gm/dl	12-16 gm/dl
Total leukocyte count	5100 cells/cumm	4500-11000 cells/cumm
Serum bilirubin (total)	1.1 mg/dl	0.1-1.2 mg/dl
Serum aspartate transaminase	32.5 IU/L	10-36 IU/L
Serum alanine transaminase	36 IU/L	7-50 IU/L
Serum alkaline phosphatase	98.5 IU/L	44-147 IU/L
POST-OPERATIVE INVESTIGATIONS		
Serum amylase	98.2 U/L	30-110 U/L
Serum lipase	86 U/L	20-120 U/L

**Figure 2:**
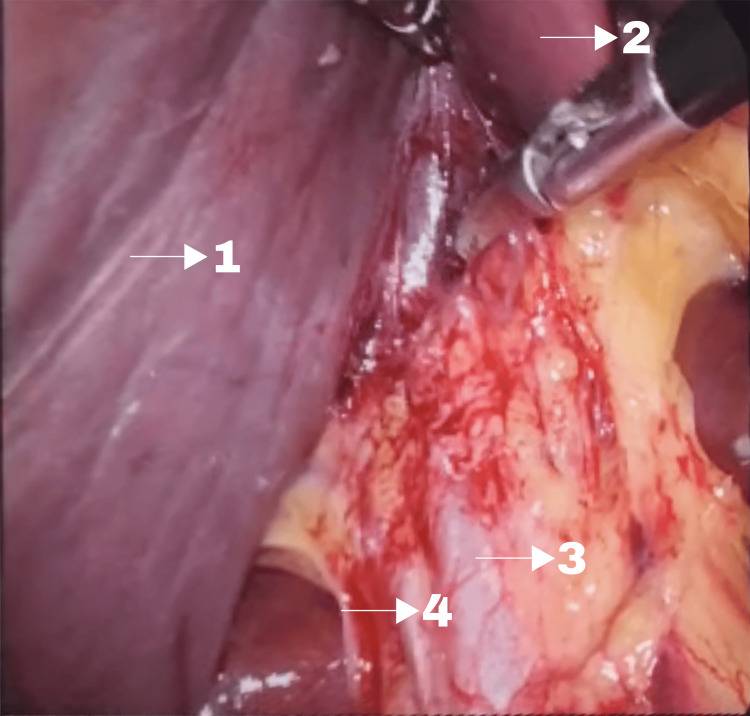
Intra-operative image revealing the absence of gallbladder. The markings labelled are enlisted as: 1. Right lobe of liver, 2. Caudate lobe of liver, 3. Common bile duct, and 4. Rouviere's sulcus.

Intraoperatively, the gallbladder could not be found even after thorough dissection in the region of porta hepatis and all possible ectopic sites, and the decision was taken to abandon the procedure (Figure 2).

**Figure 3:**
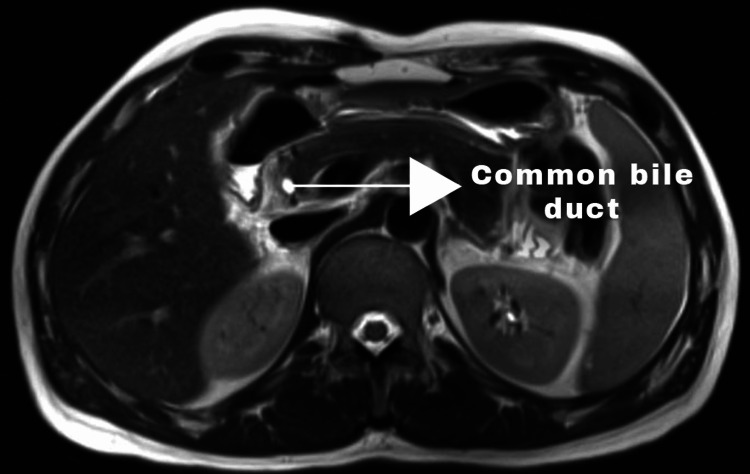
Magnetic resonance cholangiopancreatography - axial section image (done in postoperative period) - showing normal common bile duct but absence of gallbladder and cystic duct.

**Figure 4:**
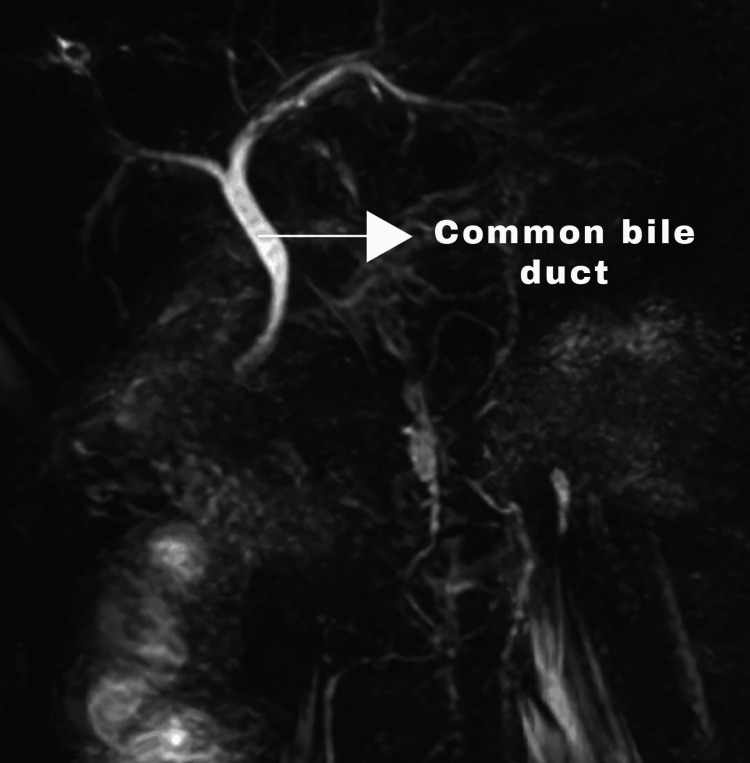
Magnetic resonance cholangiopancreatography - coronal section image - showing normal common bile duct and absence of gallbladder and cystic duct.

Postoperatively, he was evaluated by magnetic resonance cholangiopancreatography (MRCP). It showed a normal CBD with no gallbladder and cystic duct (Figures 3, 4).

Blood investigations (post-operative) such as serum amylase and lipase were also normal (Table 1). The patient was managed with analgesics and antacids. He was discharged after five days of hospitalization.

## Discussion

The hepatobiliary system development starts early in the fourth week of intrauterine life. It grows as a ventral outpouching at the lower part of the foregut. This ventral outgrowth further divides and differentiates into two parts: one as the primordium of the liver and the other representing the primordium of the gallbladder and cystic duct. Later in the development, the vacuolization of pars cystica leads to the development of the lumen of the gallbladder [3,6]. It is supposed that failure of the development of cystic bud leads to gallbladder agenesis [3]. Agenesis of the gallbladder is one of the many variations that have been described in the extrahepatic biliary system. The other not-so-common embryological development variant that needs to be discussed is an ectopic gallbladder. It occurs due to inappropriate migration of gallbladder primordium during embryonic life. Possible sites of ectopic gallbladder include intra-hepatic, left-sided, beneath the posterior inferior surface of the liver, between the leaves of the lesser omentum, within the falciform ligament, retroperitoneal, retrohepatic, or in the retropancreatic and retroduodenal areas [4,8].

Gallbladder agenesis can be associated with other congenital anomalies like horseshoe kidney, anterior abdominal wall defects, and other vascular and gastrointestinal anomalies. The symptoms of gallbladder agenesis are vague and nonspecific.

Clinically, three groups of presentation of gallbladder agenesis have been described: (1) asymptomatic (an incidental finding at laparotomy for another reason) (35%), (2) symptomatic (50%), in children with multiple fetal anomalies (such as tetralogy of fallot and agenesis of lungs), and (3) children who die in the perinatal period (15-16%).

Despite the availability of various diagnostic imaging modalities, it has been found that the preoperative diagnosis of gallbladder agenesis is still a question. Difficulty in visualization of gallbladder on routine abdominal ultrasonography should raise the suspicion of this anomaly. Magnetic resonance cholangiopancreatography (MRCP) can be done to confirm the diagnosis but is not performed routinely. Investigations like MRCP and endoscopic ultrasound (EUS) are usually done in case of any suspected diagnosis. Hepatobiliary iminodiacetic acid (HIDA) scan many times can be misleading as in the case of acute cholecystitis too the gallbladder won't be visualised.

The reported sensitivity in diagnosing gallstones on abdominal ultrasound is seen to be around 95%, but there are many other biasing factors like operator dependency and examination conditions, leading to false-positive results.

In case a diagnosis has been made in the preoperative period, conservative management in the form of muscle relaxants and antacids would suffice. Also, it has been noted that in most patients who are diagnosed intra-operatively, their symptoms resolved after nontherapeutic surgery which was true in our case. A possible explanation could be the lysis of periportal and right hypochondrial adhesions while searching for a gallbladder. However, our patient had no previous abdominal surgeries or any congenital adhesions [6].

Thorough postoperative evaluation for the symptoms must be done, considering biliary dyskinesia and sphincter of Oddi dysfunction can mimic this entity.

Conservative management is the key to the treatment of this entity. Thorough preoperative evaluation and radiological investigations in case of suspicion, can avoid unnecessary operative intervention.

## Conclusions

Agenesis of the gallbladder is a rare congenital abnormality, and a significant diagnostic challenge. With clinical and radiological features mimicking other biliary conditions, the clinician requires a high index of suspicion for preoperative diagnosis to avoid negative laparotomy. With newer advanced imaging modalities and increased awareness about this entity, this anomaly is being reported increasingly. Radiologists and clinicians should remember this diagnosis when visualization of the gallbladder is difficult on standard, routine imaging. Surgeons should approach such case scenarios more conservatively.
